# Supplementary material for: Single cell transcriptomics of human epidermis identifies basal stem cell transition states
Source: Nat Commun. 2020 Aug 25;11:4239. doi: 10.1038/s41467-020-18075-7 (PMC7447770; doi:10.1038/s41467-020-18075-7)
Supplement: Supplementary file 1 — Supplementary information [file 41467_2020_18075_MOESM1_ESM.pdf]

Single cell transcriptomics of human epidermis identifies basal stem cell transition states

Wang et al.

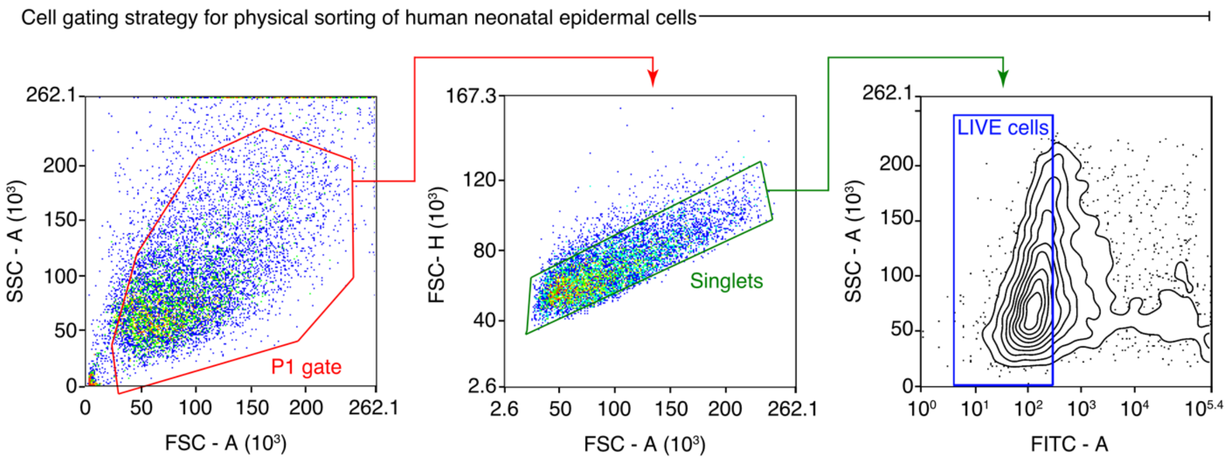

Supplementary Figure 1. Schematic of FACS strategy for physical sorting of live epidermal cells from human neonatal epidermis samples. SSC – A denotes side scatter area. FSC – A denotes forward scatter area. FSC – H denotes forward scatter height. FITC – A denotes dead cells using SYTOX Green.

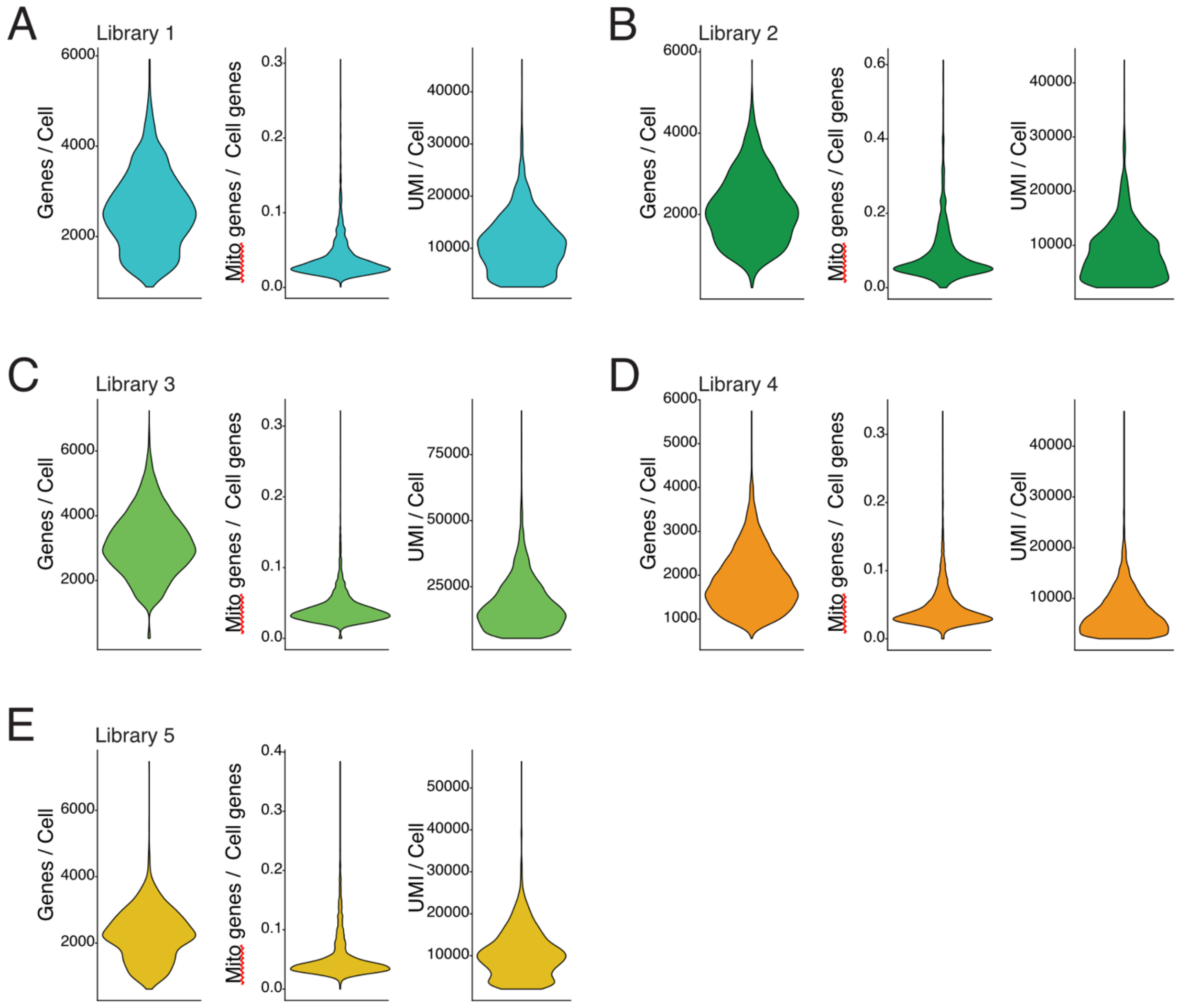

Supplementary Figure 2. Metrics of single cell libraries before quality control cutoffs. A-E) Violin plots showing genes per cell, mitochondrial (mito) genes per total cell genes, and unique molecular identifiers (UMI) per cell during quality control assessment.

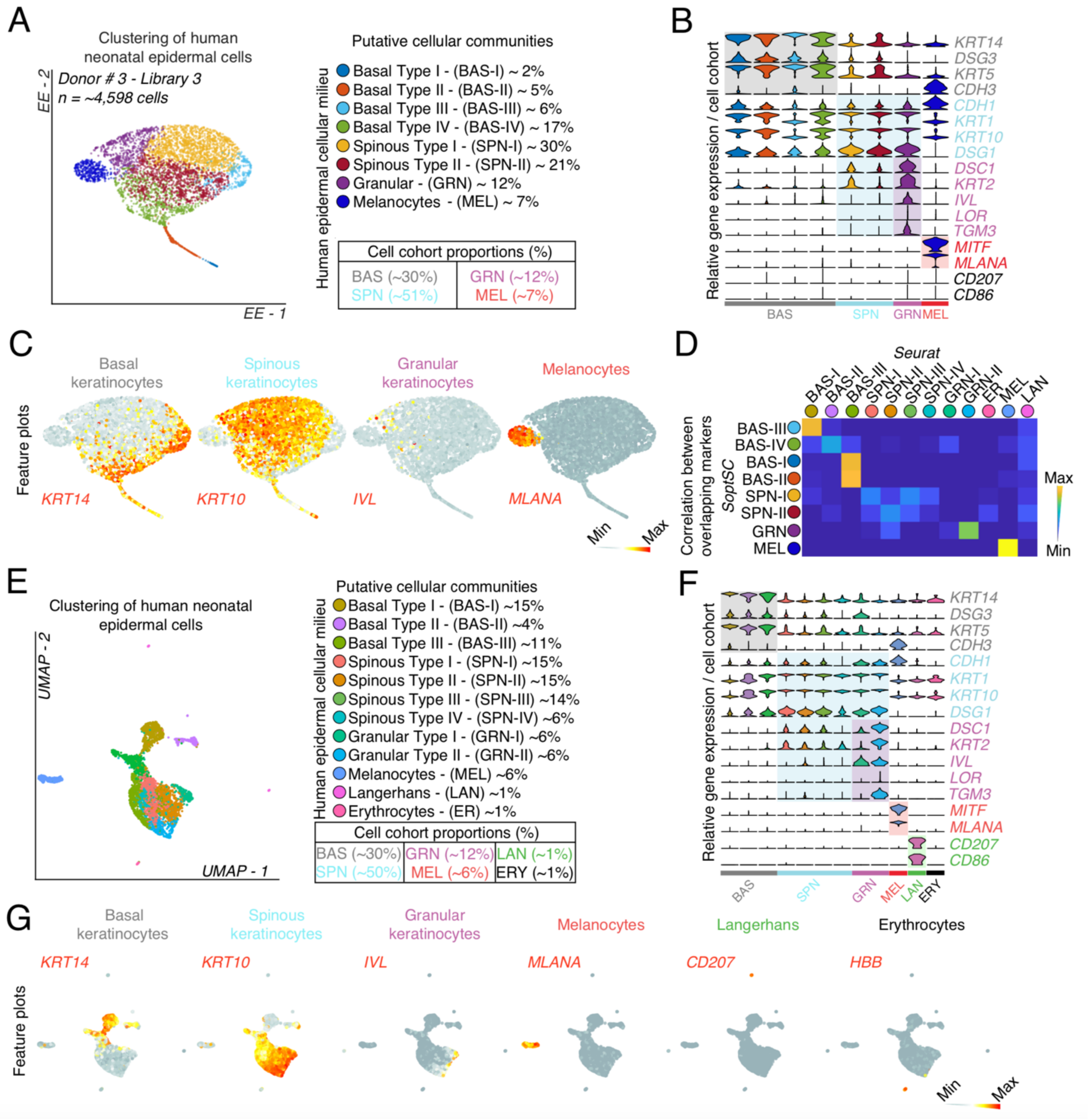

Supplementary Figure 3. Individual clustering of human neonatal epidermal cell populations. A) Clustering of 4,598 single cells isolated from Library 3 using SoptSC and displayed using Elastic Embedding (EE). Cell proportions from putative cellular communities are quantified on the right. **B)** Violin plots of relative gene expression of known epidermal marker genes split by cell cohorts using SoptSC. **C)** Feature plots showing expression of keratinocyte markers from basal, spinous, and granular layers, including melanocytes. **D)** Correlation between overlapping differentially expressed genes from SoptSC and Seurat. **E)** Clustering of 4,598 single cells isolated from Library 3 using Seurat and dimensionality reduction using UMAP. Cell proportions from putative cellular communities are quantified on the right. **F)** Violin plots of relative

gene expression of known epidermal marker genes split by cell cohorts using Seurat. **G)** Feature plots showing expression of keratinocyte markers from basal, spinous, and granular layers, including melanocytes, Langerhans cells, and erythrocytes.

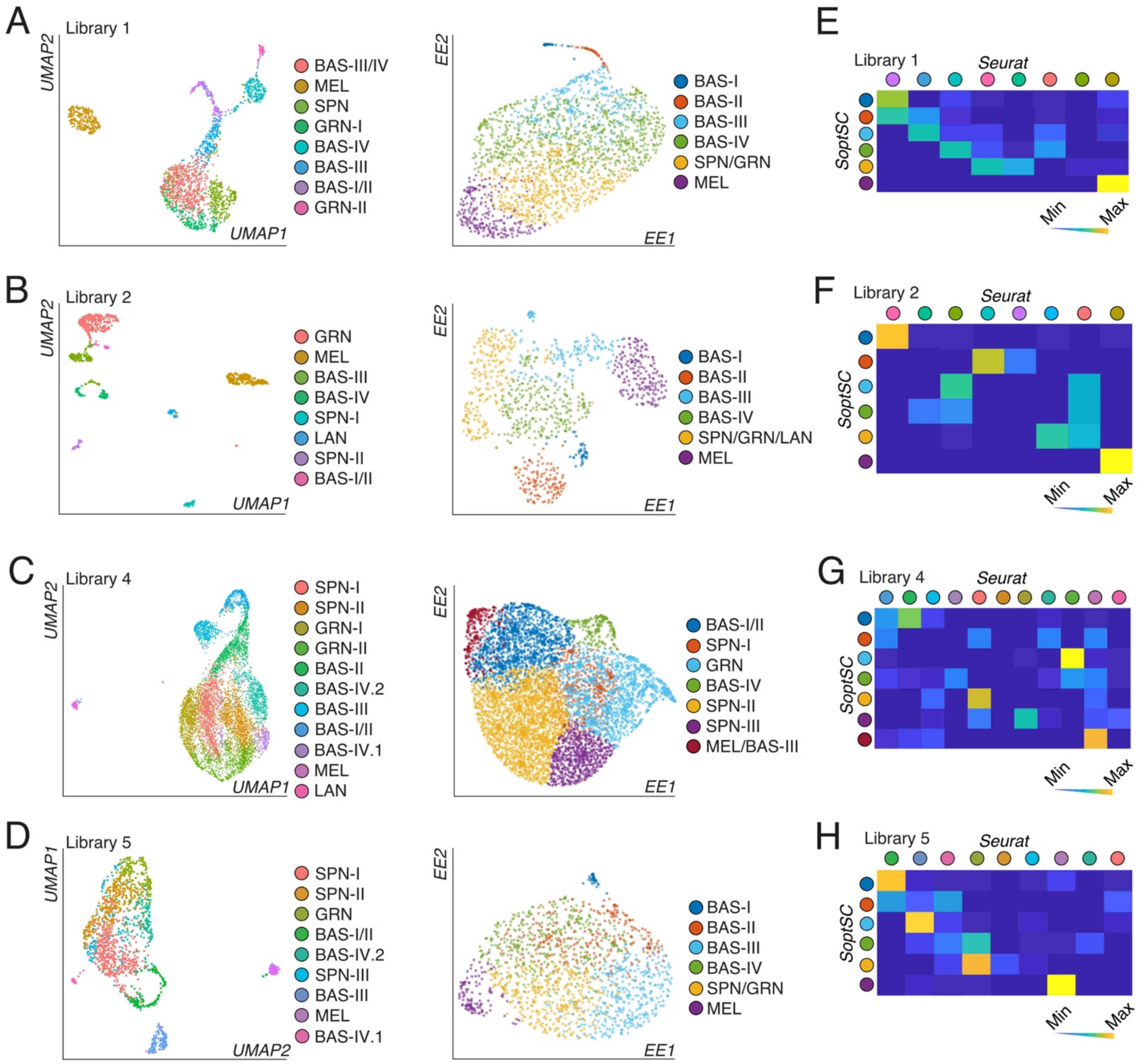

Supplementary Figure 4. Clustering and correlation analysis of epidermal cells. A-D) Clustering of single cells isolated from human neonatal epidermis using (left) Seurat and dimensionality reduction using UMAP and (right) using SoptSC and displayed using EE. Cell proportions from putative cellular communities are quantified on the right of each plot. Library numbers as labeled. **E-H)** Correlation between overlapping differentially expressed genes from SoptSC and Seurat. Library numbers as labeled.

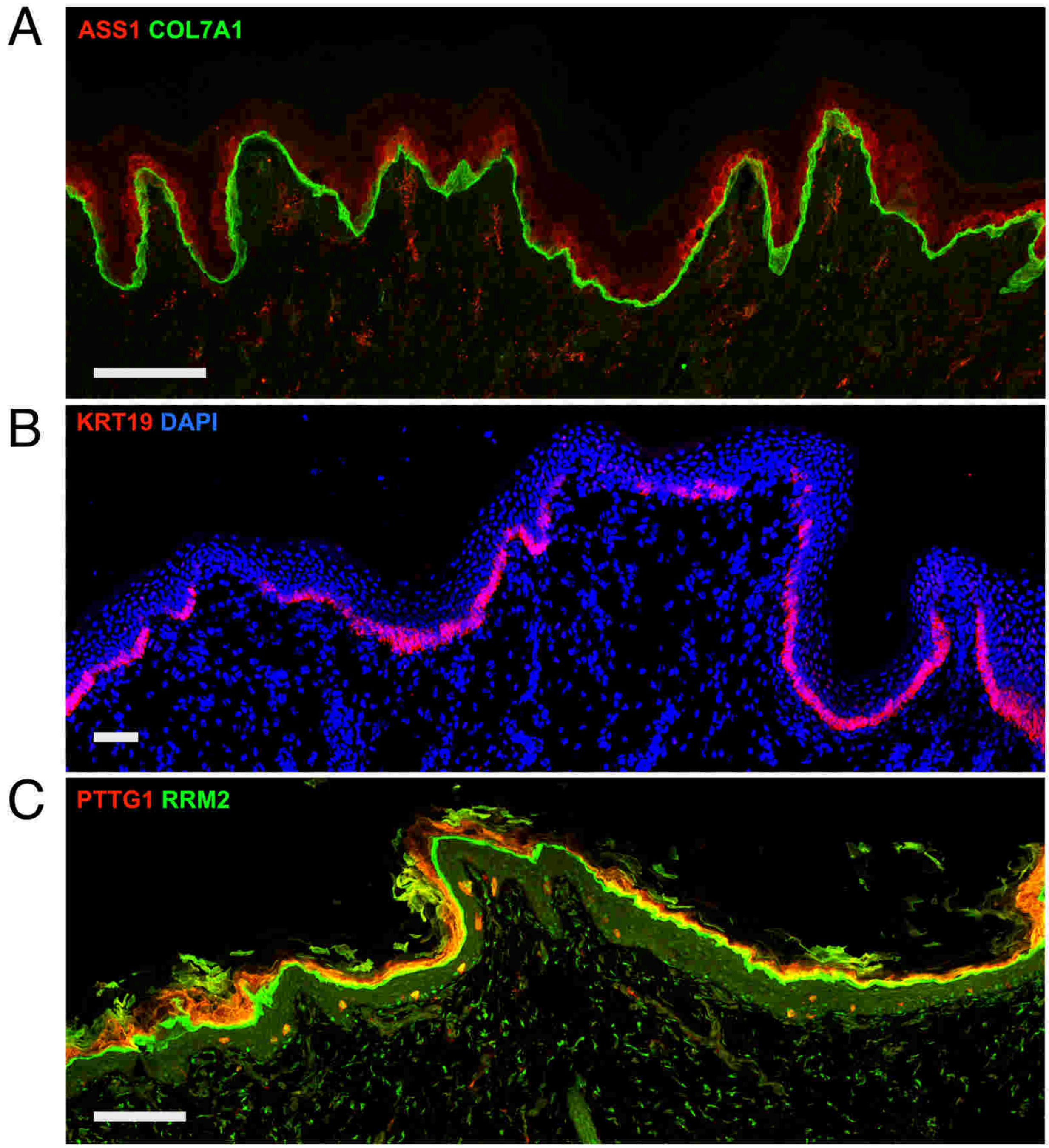

Supplementary Figure 5. Immunostaining of basal cell clusters. A) Immunostaining of ASS1 and COL17A1 (n = 6), **B)** KRT19 and DAPI (n = 3), **C)** and PTTG1 and RRM2 (n = 3) in human neonatal skin. Scale bar 100μm.

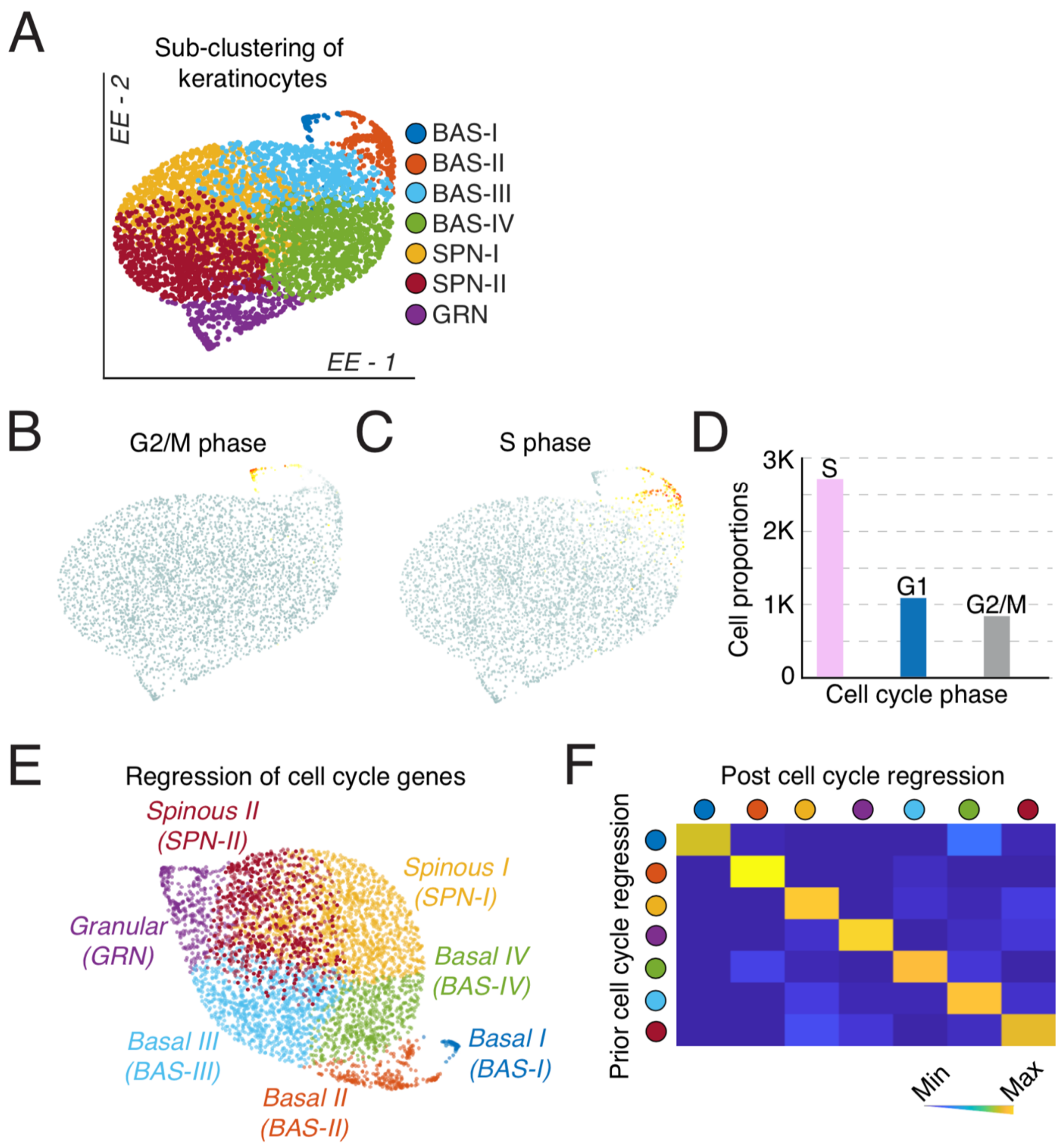

Supplementary Figure 6. Cell cycle analysis of epidermal cells. A) Subclustering of epidermal keratinocytes from Library 3 using SoptSC and displayed using EE. **B)** G2/M phase score overlaid on EE space. **C)** S phase score overlaid on EE space. **D)** Quantification of cell cycle phase state. K, 1000. **E)** Clustering of epidermal cells after regression of cell cycle genes. **F)** Correlation between overlapping markers before and after cell cycle regression.

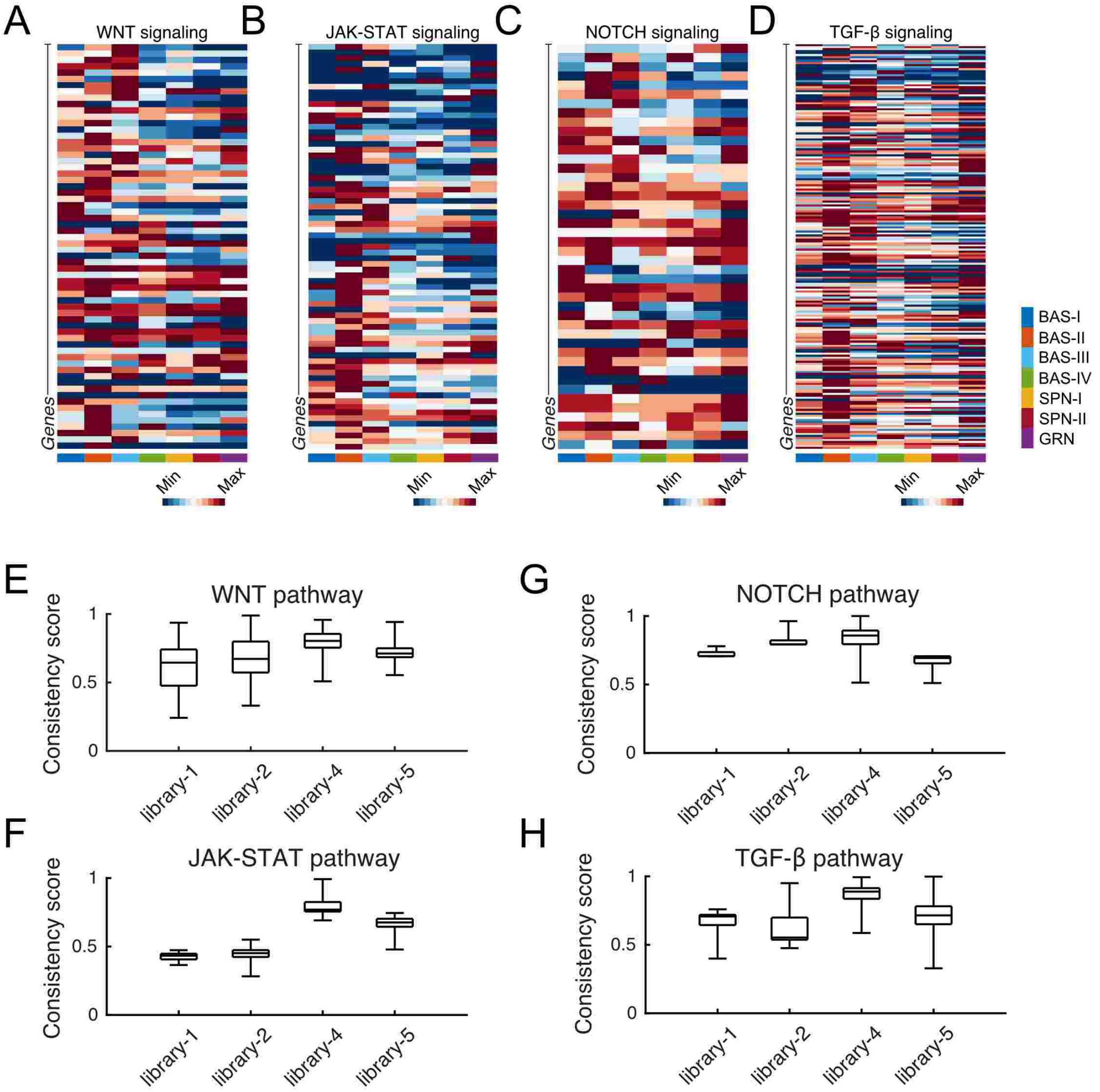

Supplementary Figure 7. Heatmaps and consistency scores of genes used to construct signaling pathways. A-D) Heatmaps showing expression levels of cognate ligand-receptor pairs and their downstream targets predicted for the **(A)** WNT, **(B)** JAK-STAT, **(C)** NOTCH, and **(D)** TGF-β signaling pathways. Left: Ligands, receptors, and their downstream target genes. **E-H)** Consistency scores of cluster-cluster signaling interactions between library 3 and the indicated libraries. For whisker and box plots: box represents 25th to 75th percentiles, whiskers represent minimum and maximum data points, and bar represents mean.

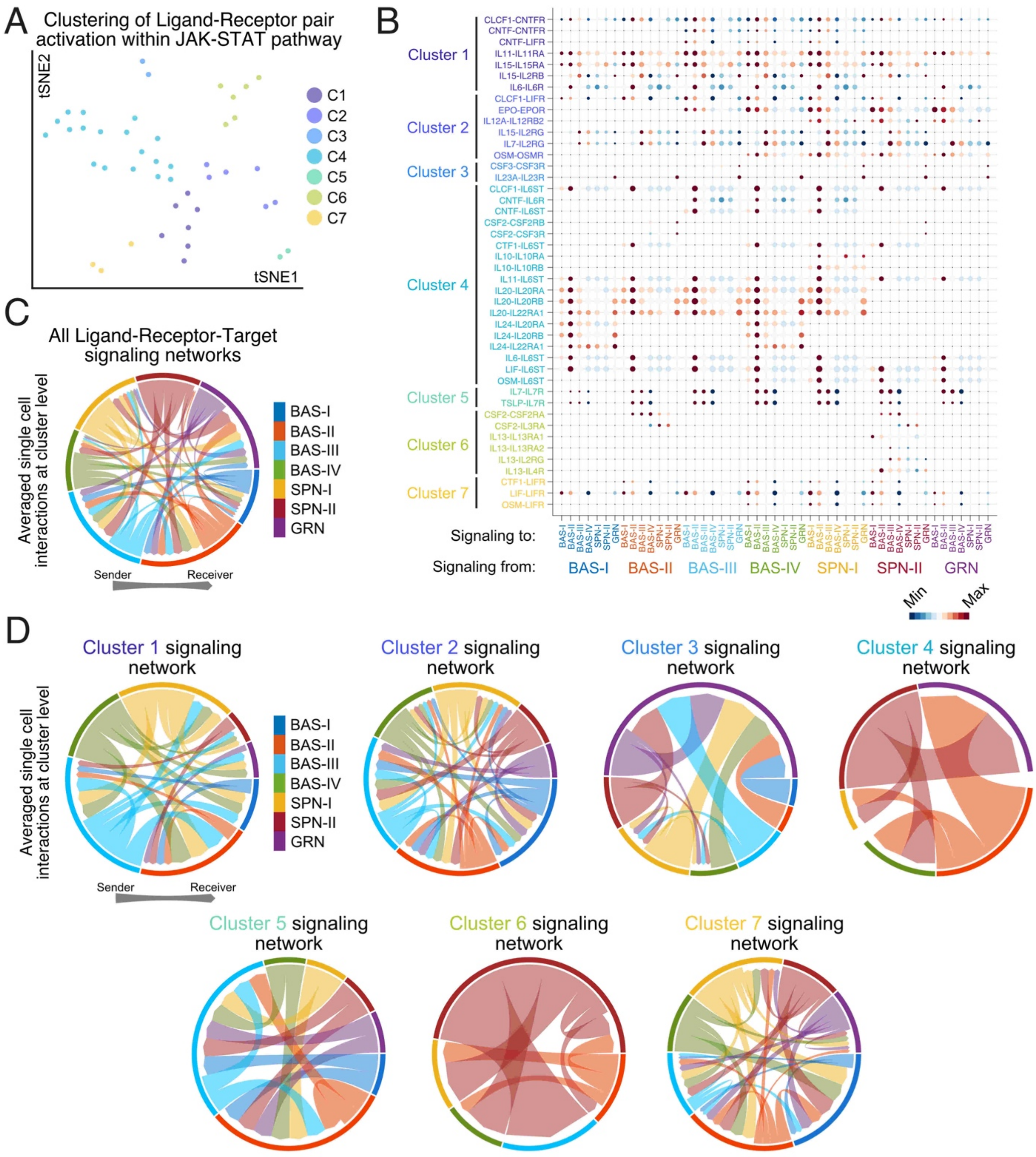

Supplementary Figure 8. Cell-cell interaction modeling of the JAK-STAT signaling pathway. A) Hierarchical clustering of similar cell-cell signaling probability scores and visualized on a tSNE plot. **B)** Visualization of signaling probability scores of Ligand-Receptor pairs and their downstream signaling components. Dot size represents number of averaged cells with probability scores between clusters. **C)** Cell-cell communication networks predicted for the entire JAK-STAT pathway or for **(D)** individual Cluster networks from B. Edge weights represent the probability of signaling between cell clusters.

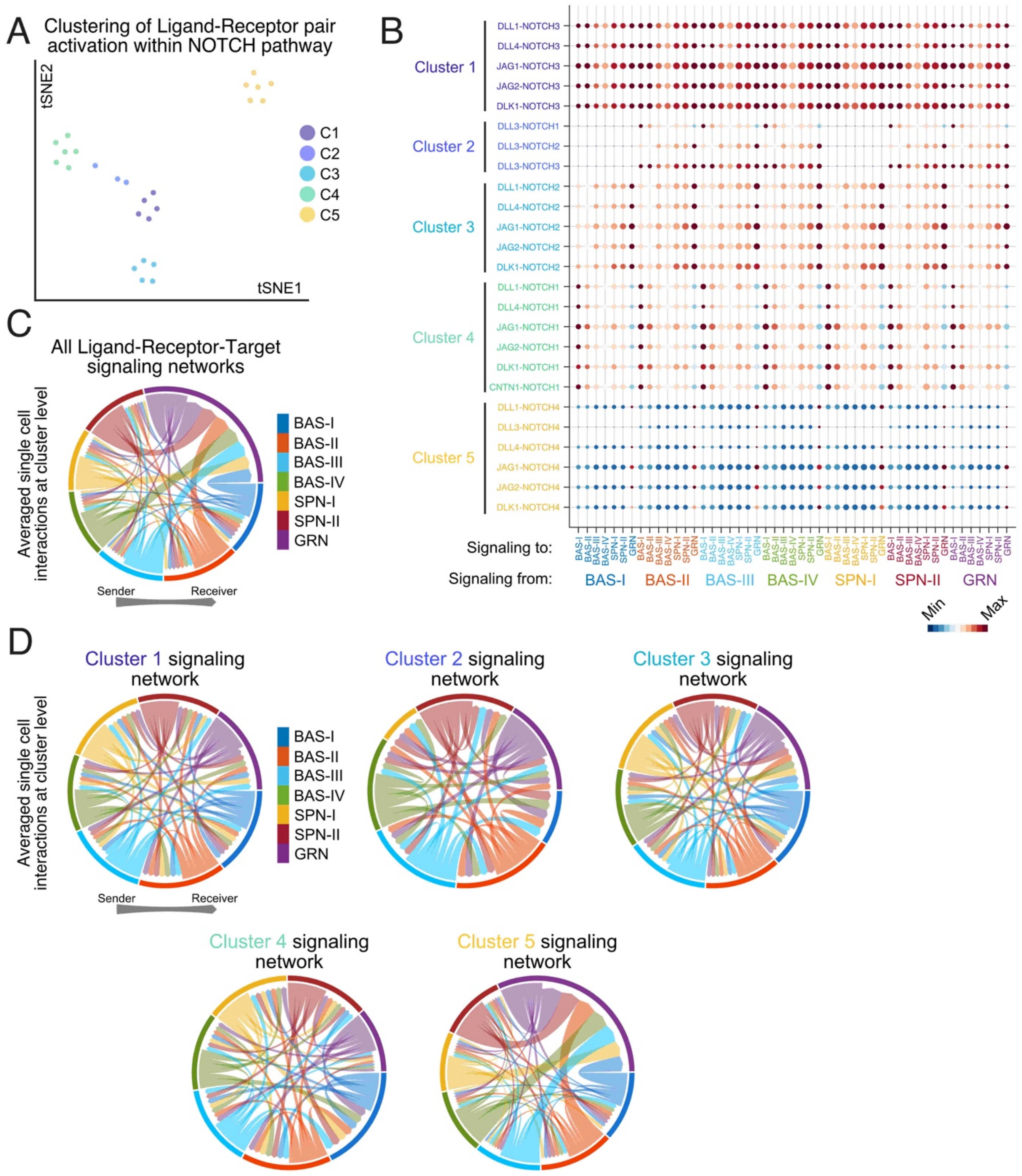

Supplementary Figure 9. Cell-cell interaction modeling of the NOTCH signaling pathway. **A)** Hierarchical clustering of similar cell-cell signaling probability scores and visualized on a tSNE plot. **B)** Visualization of signaling probability scores of Ligand-Receptor pairs and their downstream signaling components. Dot size represents number of averaged cells with probability scores between clusters. **C)** Cell-cell communication networks predicted for the entire NOTCH pathway or for **(D)** individual Cluster networks from B. Edge weights represent the probability of signaling between cell clusters.

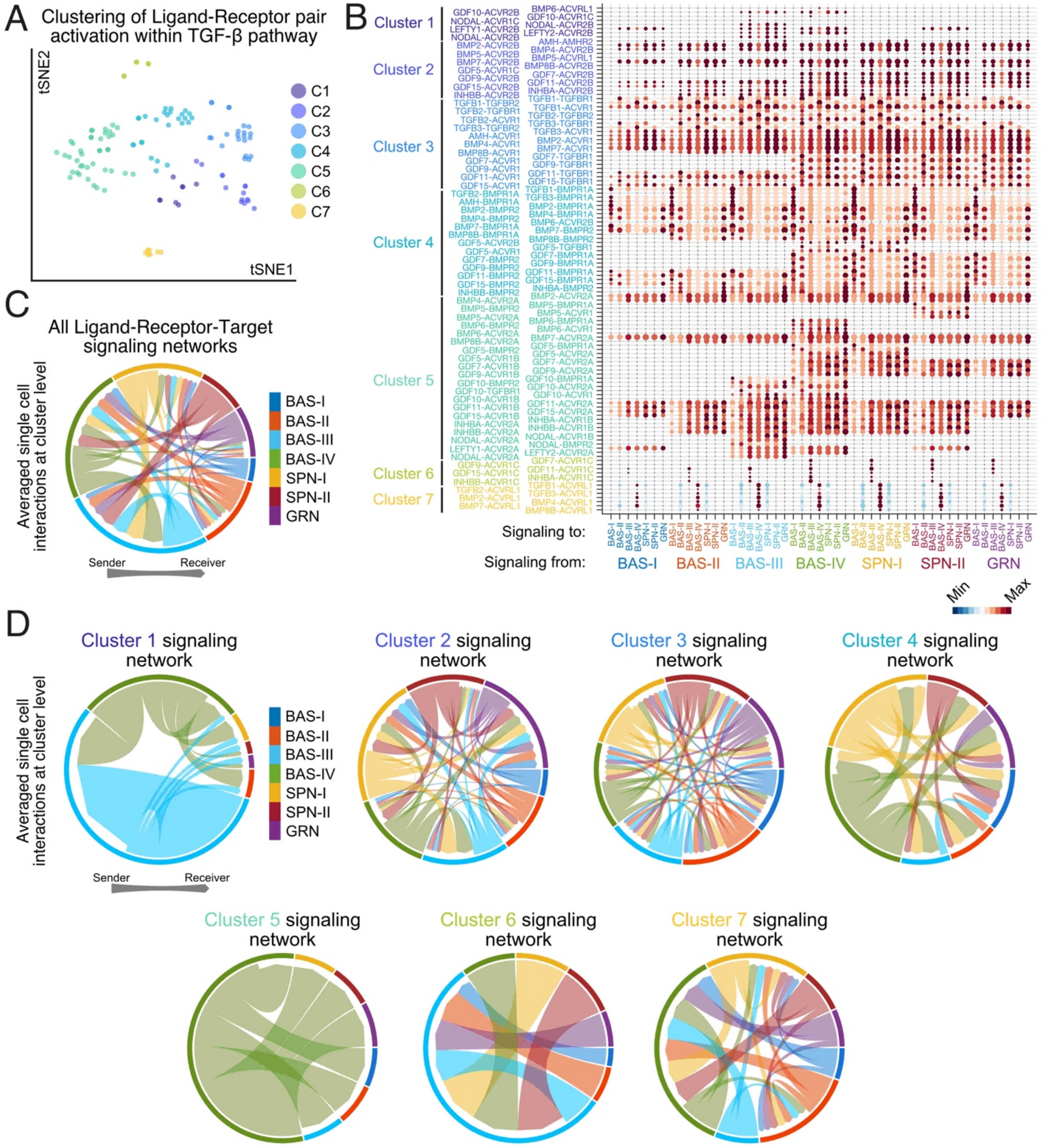

Supplementary Figure 10. Cell-cell interaction modeling of the TGF-β signaling pathway. A) Hierarchical clustering of similar cell-cell signaling probability scores and visualized on a tSNE plot. **B)** Visualization of signaling probability scores of Ligand-Receptor pairs and their downstream signaling components. Dot size represents number of averaged cells with probability scores between clusters. **C)** Cell-cell communication networks predicted for the entire TGF-β pathway or for **(D)** individual Cluster networks from B. Edge weights represent the probability of signaling between cell clusters.

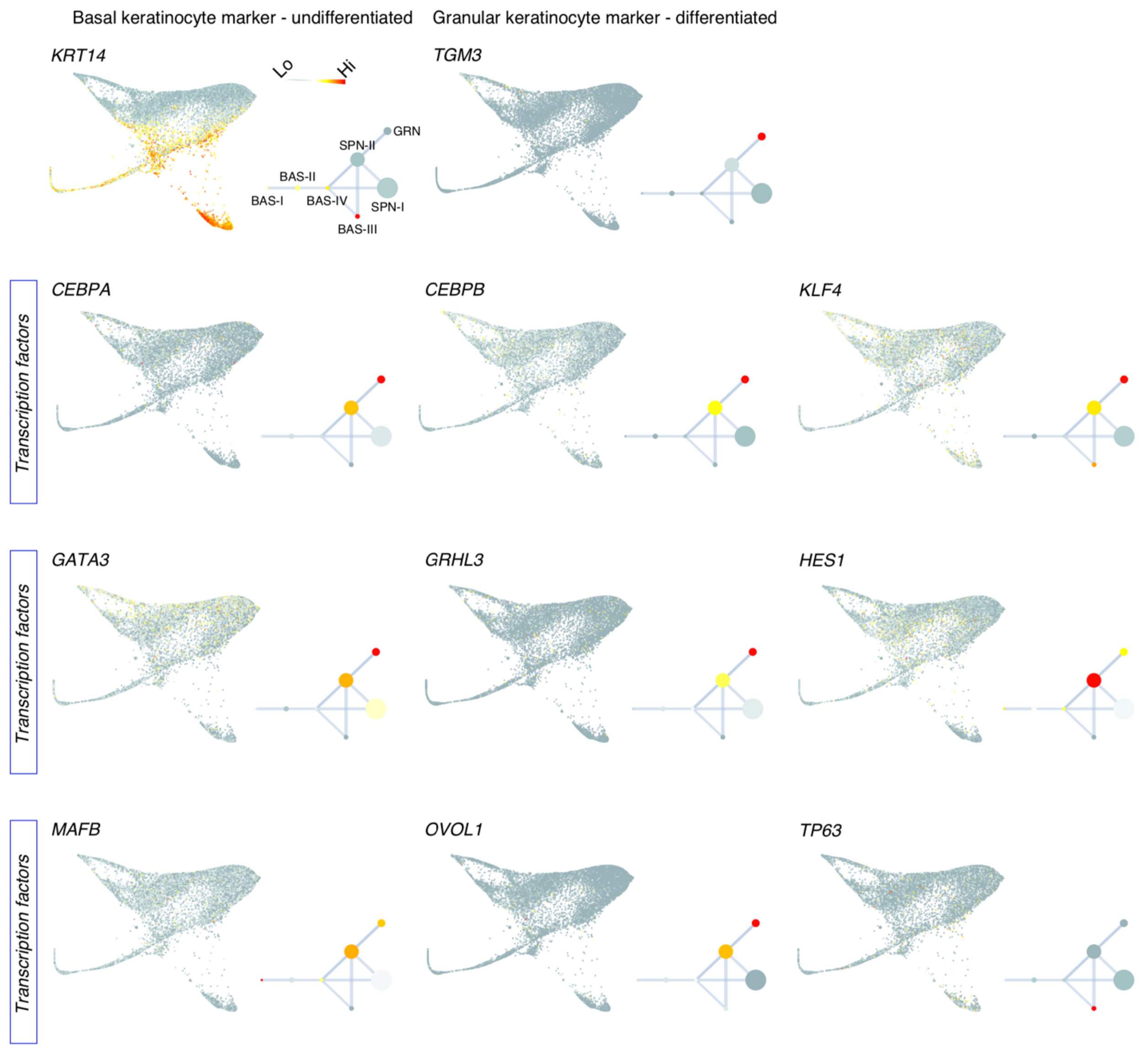

Supplementary Figure 11. Evaluation of transcription factor dynamics along pseudotime. Feature plots showing expression of select genes. Expression along the epidermal cell lineage as determined in Figure 4B displayed on the right.

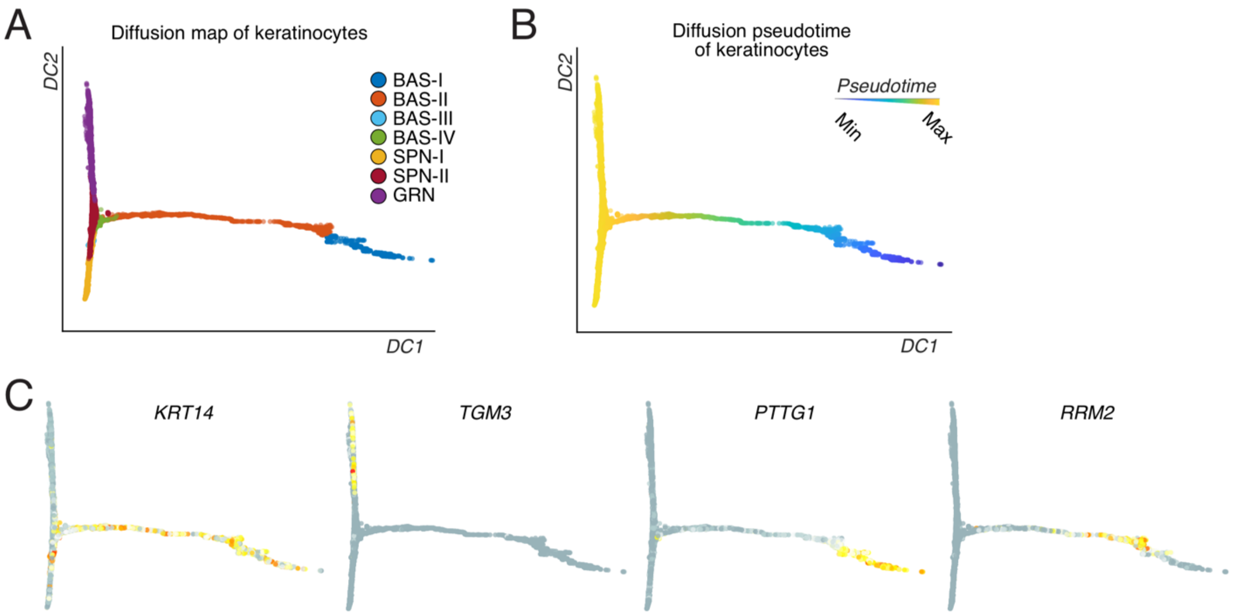

Supplementary Figure 12. Diffusion pseudotime (DPT) of keratinocytes. **A)** DPT map of keratinocytes with or **B)** without cell identities from Figure 4A superimposed. **C)** Feature plots showing expression of select genes and their expression along DPT.

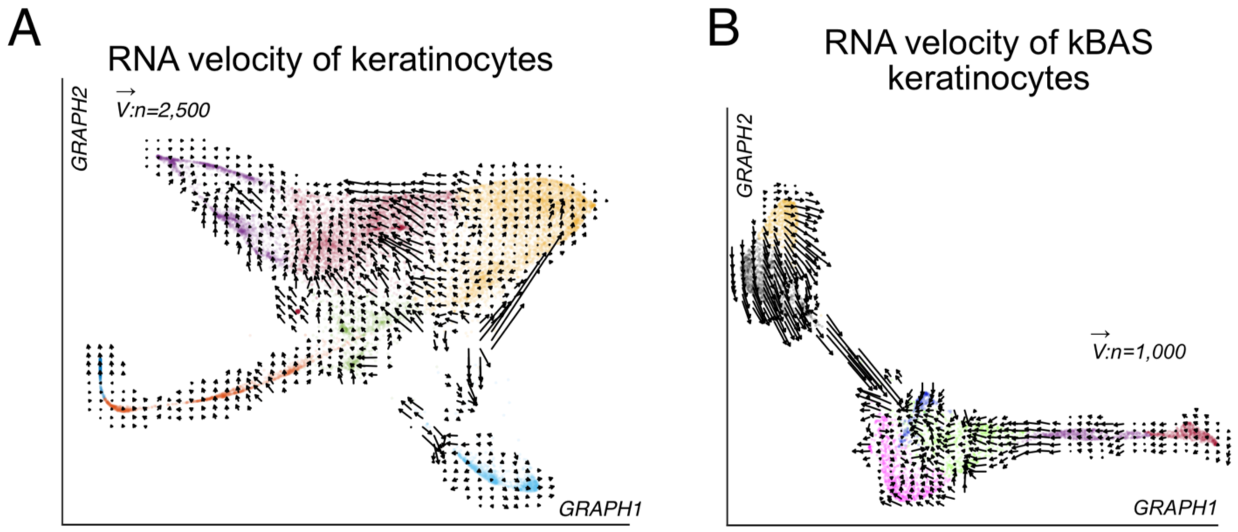

Supplementary Figure 13. Enlarged pseudotime plots from A) Figure 4E and B) Figure 5E.

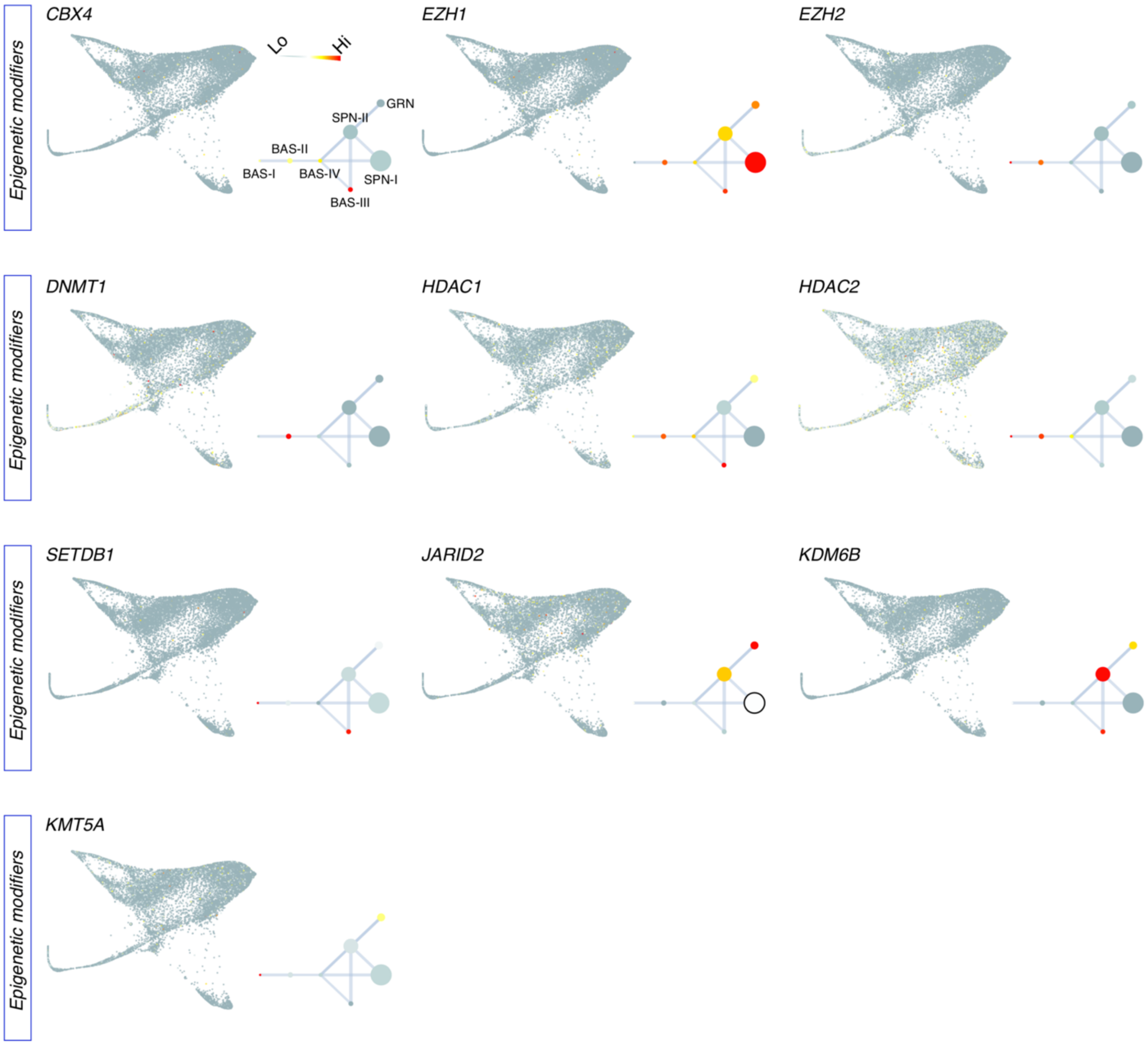

Supplementary Figure 14. Evaluation of epigenetic modifier dynamics along pseudotime. Feature plots showing expression of select epigenetic modifier genes and their expression along pseudotime. Expression along the epidermal cell lineage as determined in Figure 4B displayed on the right.

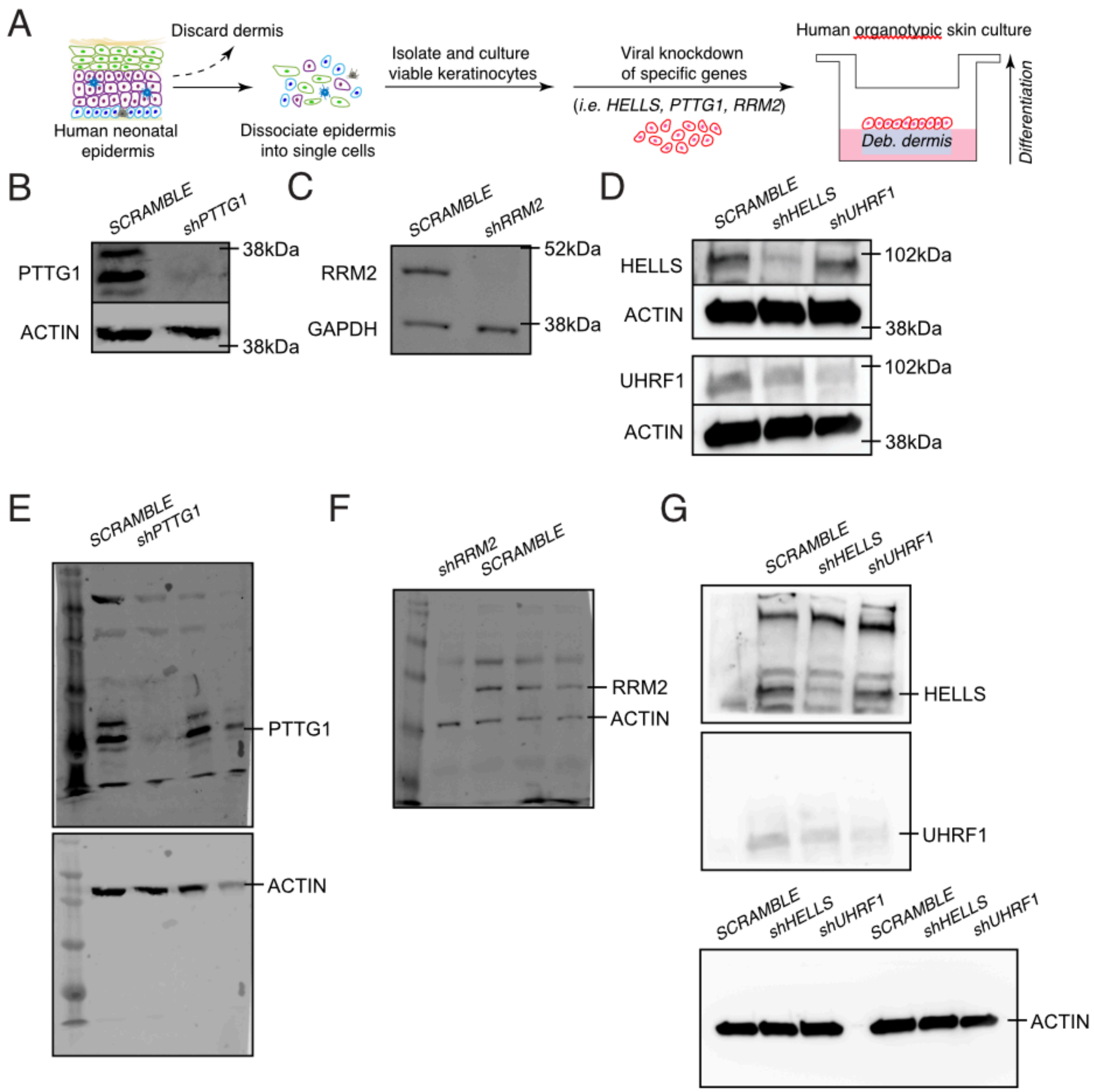

Supplementary Figure 15. Generation of human organotypic skin cultures. A) Schematic of primary keratinocyte cell isolation from human neonatal epidermis, viral knockdown, and seeding on top of devitalized human dermis at the air-liquid interface for generation of organotypic skin cultures. **B)** Western blot of PTTG1 or ACTB (ACTIN) protein in *SCRAMBLE* control or *PPTG1* knockdown (KD) (*shPTTG1*) keratinocytes. n = 4 experiments. **C)** Western blot of RRM2 or GAPDH protein in *SCRAMBLE* control or *RRM2* KD (*shRRM2*) keratinocytes. n = 4 experiments. **D)** Western blot of HELLS, UHRF1, or ACTIN protein in *SCRAMBLE* control, *HELLS* KD (*shHELLS*), or *UHRF1* KD (*shUHRF1*) keratinocytes. n = 6 experiments. **E-G)** Full scans of western blots.

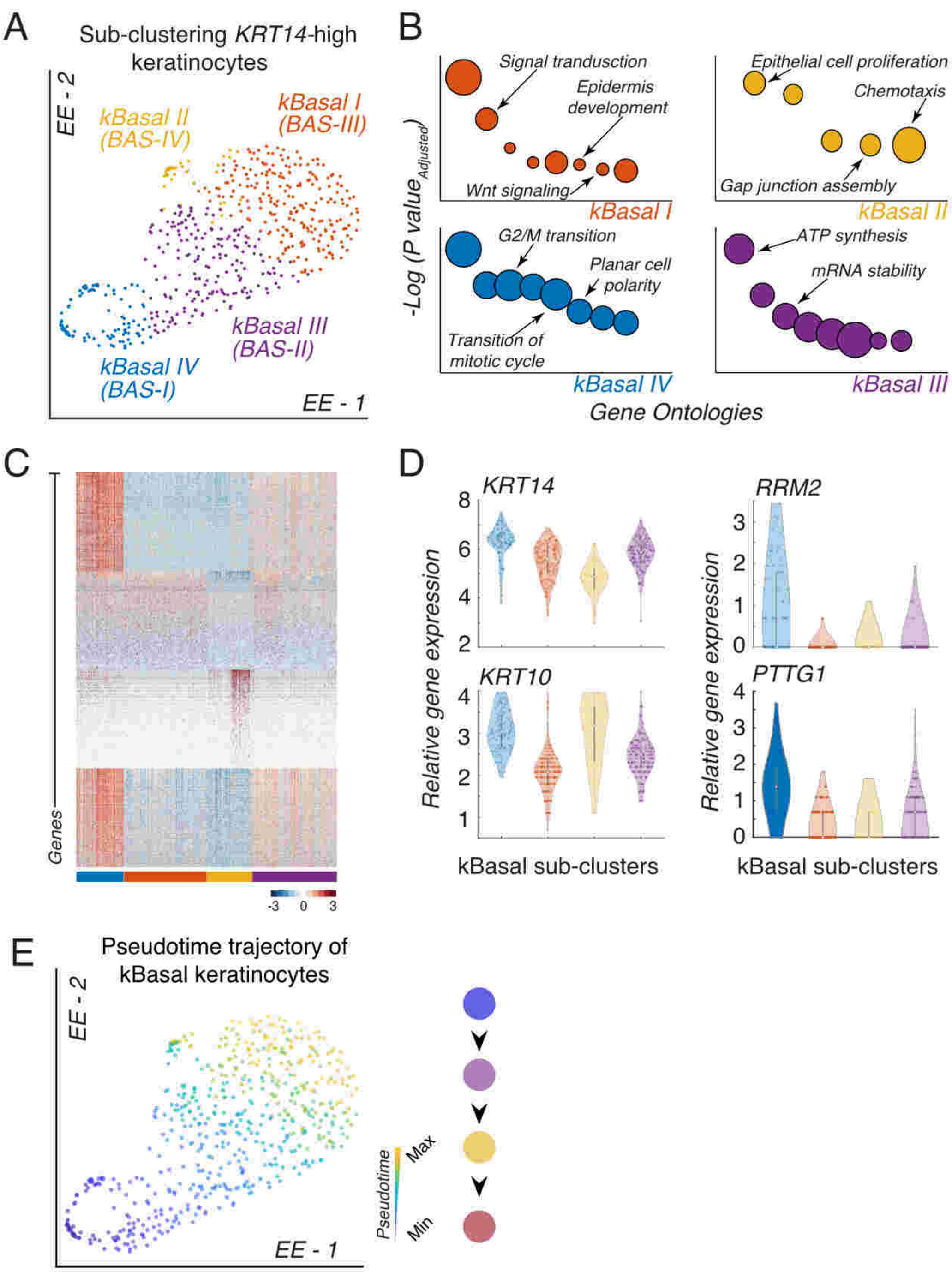

Supplementary Figure 16. Subclustering *KRT14*-high cells subsample existing basal stem cell populations with no increase in heterogeneity. A) Subclustering of *KRT14*-high keratinocytes from library 3 using SoptSC and displayed using EE. Corresponding cluster identification from Figure 5A in parenthesis. **B)** Gene ontology analysis of each kBasal cluster with selected terms labeled. **C)** Heatmap showing top 100 differentially expressed genes per cluster. Cells are color-coded as in A at the bottom. **D)** Violin plot of relative gene expression of *KRT14*, *KRT10*, *PTTG1,* and *RRM2* across the kBasal subclusters. **E)** Pseudotime inference of kbasal cluster keratinocytes displayed using EE. Cell lineage inference displayed on the right.

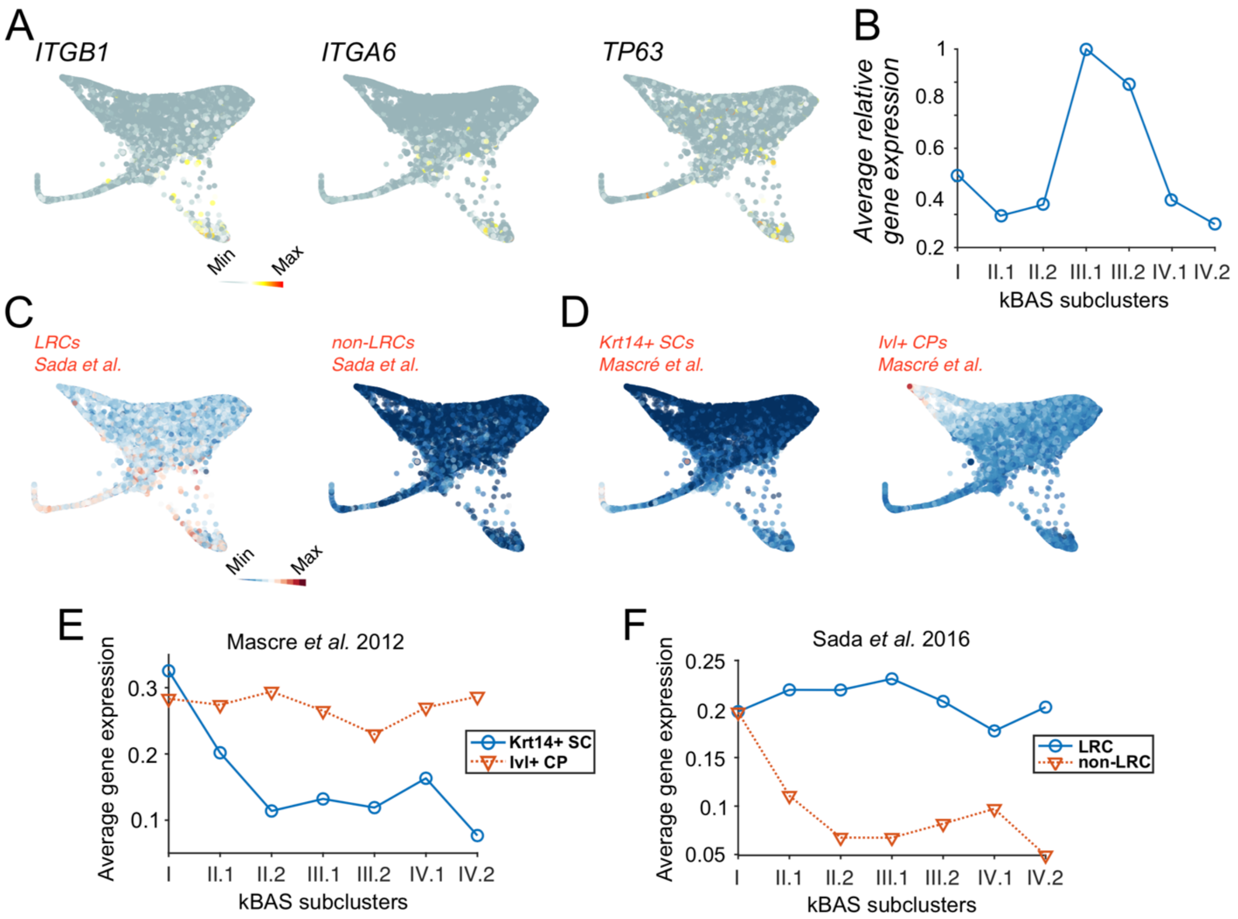

Supplementary Figure 17. Characterization of markers associated with stem cells and committed progenitors. A) Feature plots showing expression of common stem cell-associated genes (*ITGB1*, *ITGA6*, and *TP63*) overlaid on EE space. **B)** Average relative gene expression of stem cell-associated genes along the kBAS subclusters from Figure 5A. **C-F)** Feature plots and graphs showing average relative gene expression of **C, F)** label-retaining stem cells (LRC) or non-label-retaining stem cells (non-LRC)[1] or of **D-E)** *Krt14-CreER*+ (*Krt14*+) stem cells (SC) or *Ivl-CreER*+ (*Ivl*+) committed progenitors (CP)[2].

Supplementary references

1. Sada, A. *et al.* Defining the cellular lineage hierarchy in the interfollicular epidermis of adult skin. *Nature Cell Biology* **18,** 619–631 (2016).
2. Mascré, G. *et al.* Distinct contribution of stem and progenitor cells to epidermal maintenance. *Nature* (2012). doi:10.1038/nature11393
