# Supplementary material for: Single cell transcriptomics of human epidermis identifies basal stem cell transition states
Source: Nat Commun. 2020 Aug 25;11:4239. doi: 10.1038/s41467-020-18075-7 (PMC7447770; doi:10.1038/s41467-020-18075-7)
Supplement: Supplementary file 8 — Description of Additional Supplementary Files [file 41467_2020_18075_MOESM8_ESM.pdf]

Title: Supplementary Data File 1:
Description: Differentially expressed genes from integrative or single library clustering of human neonatal epidermis.

Title: Supplementary Data File 2:
Description: Ligand, receptor, and downstream target genes used for cell-cell signaling analysis of WNT, JAK/STAT, NOTCH, and TGF-β signaling pathways.

Title: Supplementary Data File 3:
Description: Differentially expressed genes from subclustering of human neonatal epidermal keratinocytes.

Title: Supplementary Data File 4:
Description: Differentially expressed genes along the putative BAS-SPN-GRN differentiation trajectory of human neonatal epidermal keratinocytes.

Title: Supplementary Data File 5:
Description: Differentially expressed genes from subclustering of human neonatal epidermal basal clusters.
